# Threshold optimization in AI chest radiography analysis: integrating real-world data and clinical subgroups

**DOI:** 10.1186/s41747-025-00632-8

**Published:** 2025-09-22

**Authors:** Jan Rudolph, Christian Huemmer, Alexander Preuhs, Giulia Buizza, Julien Dinkel, Vanessa Koliogiannis, Nicola Fink, Sophia Samira Goller, Vincent Schwarze, Maurice Heimer, Boj Friedrich Hoppe, Thomas Liebig, Jens Ricke, Bastian Oliver Sabel, Johannes Rueckel

**Affiliations:** 1 Department of Radiology, LMU University Hospital, LMU Munich, Munich, Germany; 2 XP Technology and Innovation, Siemens Healthineers AG, Forchheim, Germany; 3 Comprehensive Pneumology Center, German Center for Lung Research, Munich, Germany; 4 Department of Radiology, Asklepios Fachklinik München, Gauting, Germany; 5 Institute of Neuroradiology, LMU University Hospital, LMU Munich, Munich, Germany

**Keywords:** Artificial intelligence, Lung neoplasms, Pleural effusion, Pneumothorax, Radiography (thoracic)

## Abstract

**Background:**

Manufacturer-defined AI thresholds for chest x-ray (CXR) often lack customization options. Threshold optimization strategies utilizing users’ clinical real-world data along with pathology-enriched validation data may better address subgroup-specific and user-specific needs.

**Materials and methods:**

A pathology-enriched dataset (study cohort, 563 (CXRs)) with pleural effusions, consolidations, pneumothoraces, nodules, and unremarkable findings was analysed by an AI system and six reference radiologists. The same AI model was applied to a routine dataset (clinical cohort, 15,786 consecutive routine CXRs). Iterative receiver operating characteristic analysis linked achievable sensitivities (study cohort) to resulting AI alert rates in clinical routine inpatient or outpatient subgroups. “Optimized” thresholds (OTs) were defined by a 1% sensitivity increase leading to more than a 1% rise in AI alert rates. Threshold comparisons (OTs *versus* AI vendor’s default thresholds (AIDT) *versus* Youden’s thresholds) were based on 400 clinical cohort cases with expert radiologists’ reference.

**Results:**

AIDTs, OTs, and Youden’s thresholds varied across scenarios, with OTs differing based on tailoring for inpatient or outpatient CXRs. AIDT lowering most reasonably improved sensitivity for pleural effusion, with increases from 46.8% (AIDT) to 87.2% (OT) for outpatients and from 76.3% (AIDT) to 93.5% (OT) for inpatients; similar trends appeared for consolidations. Conversely, regarding inpatient nodule detection, increasing the threshold improved accuracy from 69.5% (AIDT) to 82.5% (OT) without compromising sensitivity. Graphical analysis supports threshold selection by illustrating estimated sensitivities and clinical routine AI alert rates.

**Conclusion:**

An innovative, subgroup-specific AI threshold optimization is proposed, automatically implemented and transferable to other AI algorithms and varying clinical subgroup settings.

**Relevance statement:**

Individually customizing thresholds tailored to specific medical experts’ needs and patient subgroup characteristics is promising and may enhance diagnostic accuracy and the clinical acceptance of diagnostic AI algorithms.

**Key Points:**

- Customizing AI thresholds individually addresses specific user/patient subgroup needs.
- The presented approach utilizes pathology-enriched and real-world subgroup data for optimization.
- Potential is shown by comparing individualized thresholds with vendor defaults.
- Distinct thresholds for in- and outpatient CXR AI analysis may improve perception.
- The automated pipeline methodology is transferable to other AI models or subgroups.

**Graphical Abstract:**

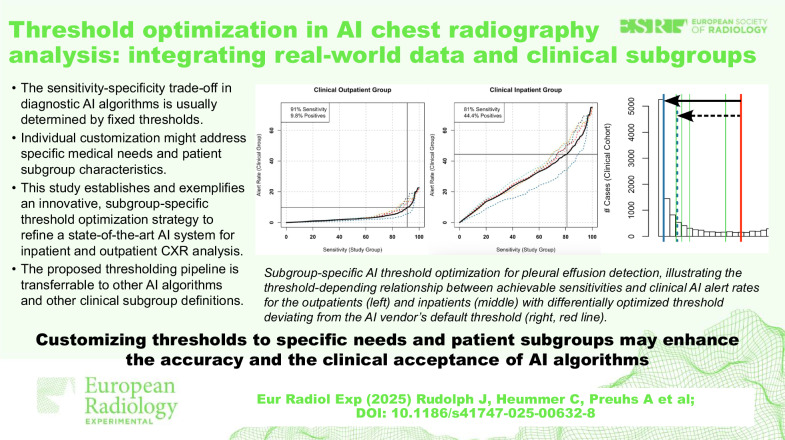

## Background

Chest x-ray radiography (CXR) analysis using artificial intelligence (AI) has gained significant attention and is being increasingly integrated into clinical practice [1–9]. Pathology detection relies on algorithmic confidence scores, which are assumed to correlate with the likelihood of underlying diseases. Preclinical algorithm training and validation commonly use large public datasets, often with mixed-quality annotations, sometimes generated through automated report interpretation via natural language processing [10–13]. Smaller clinical datasets, on the other hand, may require artificial enrichment to represent less common pathologies adequately [3, 14–18].

Receiver Operating Characteristic (ROC) analysis, a standard method for evaluating medical tests [19], offers two key advantages: it assesses binary classification (pathology yes/no) across various thresholds [20], and its area under the curve (AUC) is independent of pathology prevalence [21], making it suitable for pathology-enriched validation datasets. However, while ROC analysis is useful scientifically, it is less applicable to clinical decision-making, which demands calibrated, interpretable outputs. Selecting and optimizing cutoff thresholds to convert algorithmic scores into binary decisions is critical for clinical adoption [22, 23]. This tradeoff between sensitivity and specificity, however, is typically predefined by algorithm developers or vendors and may not be user-adjustable for specific clinical subgroups. Furthermore, its associated cutoff thresholds are often derived from artificially composed (*e.g*., pathology-enriched) cohorts with prevalence-independent optimization strategies applied (*e.g*., according to Youden’s J criterion) without taking the influence of real-world prevalences into account. In real-world clinical settings, different patient populations may require different thresholds, *e.g*., more sensitive ones, even at the cost of false positives, depending on factors like hospitalization status, comorbidities or typical pathology co-occurrences. Hence, AI threshold optimization strategies should be able to be tailored to clinical environments where diverse conditions and patient populations are represented, rather than solely relying on vendor-defined, static thresholds or being solely derived from enriched datasets.

Despite the importance of possibly adaptive thresholds, most validation studies fail to extend the analysis beyond ROC assessments, thereby neglecting the differential exploration of threshold optimization potential for specific clinical subgroups. This study introduces an innovative pipeline for subgroup-specific threshold optimization, based not only on a pathology-enriched dataset, but also on a real-world 1-year cohort of 15,786 consecutive CXRs from a major European university hospital. A proposed threshold optimization strategy, which additionally incorporates real-world data, finally enables a threshold optimization analysis that can differentially consider clinical subgroups, here with an exemplary emphasis on in- and outpatients. The detection of four key pathologies (pneumothorax, pleural effusion, pneumonia-suspected consolidations, and nodules) is targeted with underlying expert radiologist’s reference assessments; the optimization approach is exemplarily demonstrated for a state-of-the-art AI system (though readily applicable to other systems). We hypothesize that threshold optimization potential can be identified differentially for clinical inpatients and outpatients, in comparison to thresholds traditionally optimized using Youden’s J Index and/or those provided by the AI algorithm vendor.

## Materials and methods

### Ethics

Approval of the institutional ethics committee (Ethics Committee of the Medical Faculty of Ludwig-Maximilians-University Munich) was obtained for this study (approval number 19-541). Informed consent was waived due to the retrospective character of the study.

### CXR cohorts and expert medical reading

Two different cohorts (“study cohort” and “clinical cohort,” as described below) have been used for this study. Included cases (CXRs acquired in patients’ upright position only) were identified by text research based on radiology reports and/or by the date of image acquisition. Posterior-anterior projections of included cases have been exported as “Digital Imaging and Communication in Medicine” (DICOM) files, anonymized and analyzed by the AI algorithm introduced below.

The pathology-enriched study cohort was an artificially composed cohort of 563 CXRs from the emergency department of a primary care hospital. Details of this established cohort, including a comprehensive description, have been previously published [4, 17, 24, 25]. The dataset encompasses CXRs acquired between 2000 and 2018 and has been preselected with the intention to achieve balanced prevalences (10–20%) of the considered pathologies (no suspected pathologies at all, pleural effusions, pneumothoraces, consolidations suspicious for pneumonia and nodules). The images were subject to independent assessments by six different radiology professionals: three board-certified radiologists (BCRs) with 17, 9, and 7 years of experience in thoracic imaging, and three experienced radiology residents (RRs) with 4, 3, and 2 years of expertise in chest radiography imaging (readers received comprehensive reading instructions both verbally and in writing). The reference readers evaluated the images for the presence of the above-mentioned pathologies utilizing a 5-point Likert scale: 0 (no suspicion); 1 (unlikely); 2 (possible); 3 (likely) and 4 (certain finding). With regard to nodules, readers were instructed to consider nodules of various entities, including, *e.g*., benign granulomas. Additionally, if a nodule was identified, readers were prompted to assess whether they deemed an additional computed tomography scan necessary. Only these latter (clinically relevant) nodules were finally considered.

The clinical cohort encompassed all upright CXRs acquired in 2018 (*n* = 15,786) within the same university hospital that could be clearly categorized into inpatient (*n* = 11,900) or outpatient (*n* = 3,619) examinations based on available image metadata (Table 1). A small number of 267 CXRs was excluded due to the inability to determine or unequivocally classify their inpatient or outpatient status. 200 randomly selected inpatient CXRs and an additional 200 randomly chosen outpatient CXRs underwent assessment by another independent BCR who was not involved in the reading of the pathology-enriched study cohort. This BCR expert reader possessed experience in AI-based chest radiography analysis and was specifically instructed to discern which CXRs should be identified by an AI algorithm sensitized for the detection of pleural effusions, consolidations suspicious for pneumonia, pneumothoraces and clinically relevant nodules.

**Table 1:** Cohorts’ characteristics and pathology prevalence

Cohort	Study cohort	Clinical cohort	
Number (*n*)	563	15,786	
Age (mean ± standard deviation)	49.9 ± 19.3 years	61.0 ± 16.8 years	
Females (*n*, (%))	244 (43%)	6,336 (40.1%)	
Timeframe of image acquisition	2000–2018	2018 (all upright chest radiographs from that year)	
Patient subgroups (n, (%))	Outpatients 563 (100%)	Outpatients 3,619 (22.9%) Inpatients 11,900 (75.4%) Partial inpatients 157 (1.0%) Not classified 110 (0.6%)	
Pathology prevalences	Pathology-enriched, calculated prevalences according to BCR reading consensus and resulting most sensitive reference standard (RFS) IV as previously described by Rudolph et al [4, 17, 24]	Prevalence estimation according to sample reading of 200 cases each from the inpatient and outpatient groups, numbers (*n*) are calculated according to the expected prevalence	
	**RFS IV**	**Outpatient group**	**Inpatient group**
Pleural effusion (*n*, (%), 95% CI)	140 (24.9%)	851 (23.5%, 17.6–29.4%)	5,534 (46.5%, 39.6–53.4%)
Consolidation (*n*, (%), 95% CI)	146 (25.9%)	851 (23.5%, 17.6–29.4%)	3,451 (29.0%, 22.7–35.3%)
Pneumothorax (*n*, (%), 95% CI)	58 (10.3%)	91 (2.5%, 0.3–4.7%)	1,488 (12.5%, 7.9–17.1%)
Nodules (*n*, (%), 95% CI)	47 (8.4%)	290 (8.0%, 4.2–11.8%)	655 (5.5%, 2.3–8.7%)

*CI* Confidence interval, *BCR* Board-certified radiologists, *RFS* Reference standard

### AI algorithm

The tested AI algorithm is designed as a single-shot object detection neural network with a residual backbone, a convolutional feature pyramid network, and a discriminator network [26]. It is trained using stochastic gradient descent to jointly classify and detect image regions suspicious for pathological findings by minimizing a loss function that accounts for multi-label classification (based on the focal loss) and coordinate regression tasks (based on region overlaps and center distances). Regarding pneumothorax detection, manually annotated supine chest radiograph datasets have been incorporated in algorithm training, which helped to mitigate the known bias of incorrectly detecting thoracic drains as pneumothorax [27, 28]. The fully convolutional architecture processes the entire image that is resized to a shape of 1,025 × 1,025 (bilinear interpolation, aspect ratio is preserved) with pixel values between 0 and 1 (quantile-based linear mapping). During training, image augmentation techniques (*e.g*., left/right flip, cropping, rotations) are used to further increase the generalization capabilities of the neural network. Details on the algorithm architecture, including the underlying training data—which align with the “Model” and “Training” sections of the 2024 updated CLAIM guideline [29]—are described in detail by Rudolph et al [4, 24]. No images from the cohorts used in this study—neither the study cohort nor the clinical cohort—were included in the training of the algorithm.

### Target sensitivity definition and threshold optimization strategy

Reducing the threshold of an AI algorithm for pathology detection evidently enhances both the sensitivity of pathology detection and the proportion of CXRs that will be labeled “positive” for the investigated pathology by the AI in clinical practice (= alert rate), supposedly concomitant with a reduction in specificity and an increase in false positives. For this study, the target sensitivity was defined as the point at which any further sensitivity increase, achieved by further lowering the AI threshold, results in a disproportionate rise in the alert rate in clinical practice. Specifically, below this “optimized” threshold (OT), each additional one percent increase in sensitivity leads to a greater than one percent increase in the alert rate in clinical routine. This novel criterion was established through internal expert consensus, recognizing that at the latest beyond this point, the algorithm would no longer demonstrate meaningful diagnostic discrimination. If any further gain in sensitivity comes at the cost of an equally high or even greater increase in alert rate, lowering the threshold further does not provide clinical benefit. Therefore, this sensitivity level and its corresponding OT were considered to represent the upper limit of a maximally justifiable and potentially still meaningful algorithm sensitization.

### Statistical analysis

The AI algorithm performance in the enriched study cohort was assessed through ROC analysis, utilizing the evaluations of six radiology professionals as distinct reference standards. This resulted in the generation of six ROC curves reflecting different performance measures (Figs. 2, 3, top row). To generate binary reference standards (RFS) for ROC analysis, the reference readers’ 5-point Likert scale has been previously pooled as follows: The most specific RFS I grouped scores 0 to 3 as negative and considered only score 4 as positive; conversely, in the most sensitive RFS IV, scores 1 to 4 were designated as positive, with 0 being negative; the intermediate RFSs II/III were formed correspondingly. This RFS pooling procedure was established through previous studies [4, 17, 24]. In the sensitizing context (see optimization strategy above), the focus for the results presented in the main part of the manuscript shifted to the most sensitive RFS IV (alternative results based on RFS I–III are available in the Supplementary material).

**Fig. 1:**
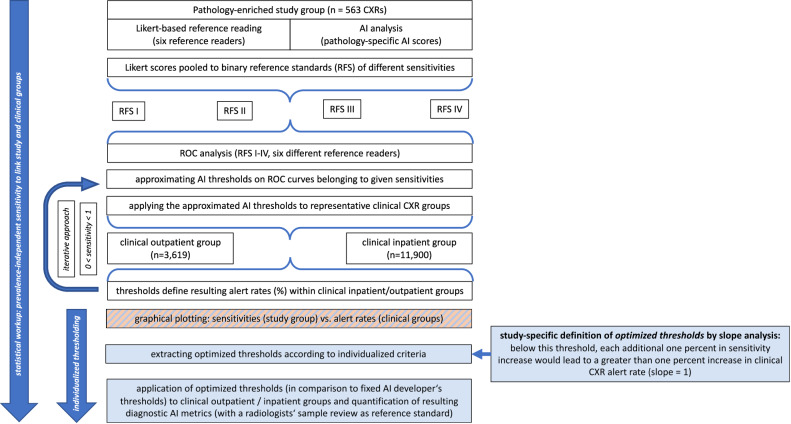
Flowchart of the pipeline for AI threshold optimization. The process begins with the pathology-enriched study group (*n* = 563 chest x-rays) evaluated by Likert-based expert readings, which are pooled to binary reference standards (RFS I–IV) with varying sensitivity levels for the following ROC analysis. A clinical cohort, including an outpatient group (*n* = 3,619) and an inpatient group (*n* = 11,900), is integrated to link sensitivities (derived from study group ROC analysis) with resulting AI alert rates (clinical subgroups) by iteratively applying different thresholds. Optimized thresholds according to the study-specific definition are derived from a slope analysis (see Figs. 2, 3, bottom row). Below these thresholds, each additional percentage increase in sensitivity results in a greater than one percent increase in AI alert rates in clinical routine. OPOT and IPOT are applied to clinical cohorts and compared with fixed AIDT. AIDT, Artificial intelligence developer’s thresholds; IPOT, Inpatient group optimized threshold; OPOT, Outpatient group optimized threshold; ROC, Receiver operating characteristic

**Fig. 2:**
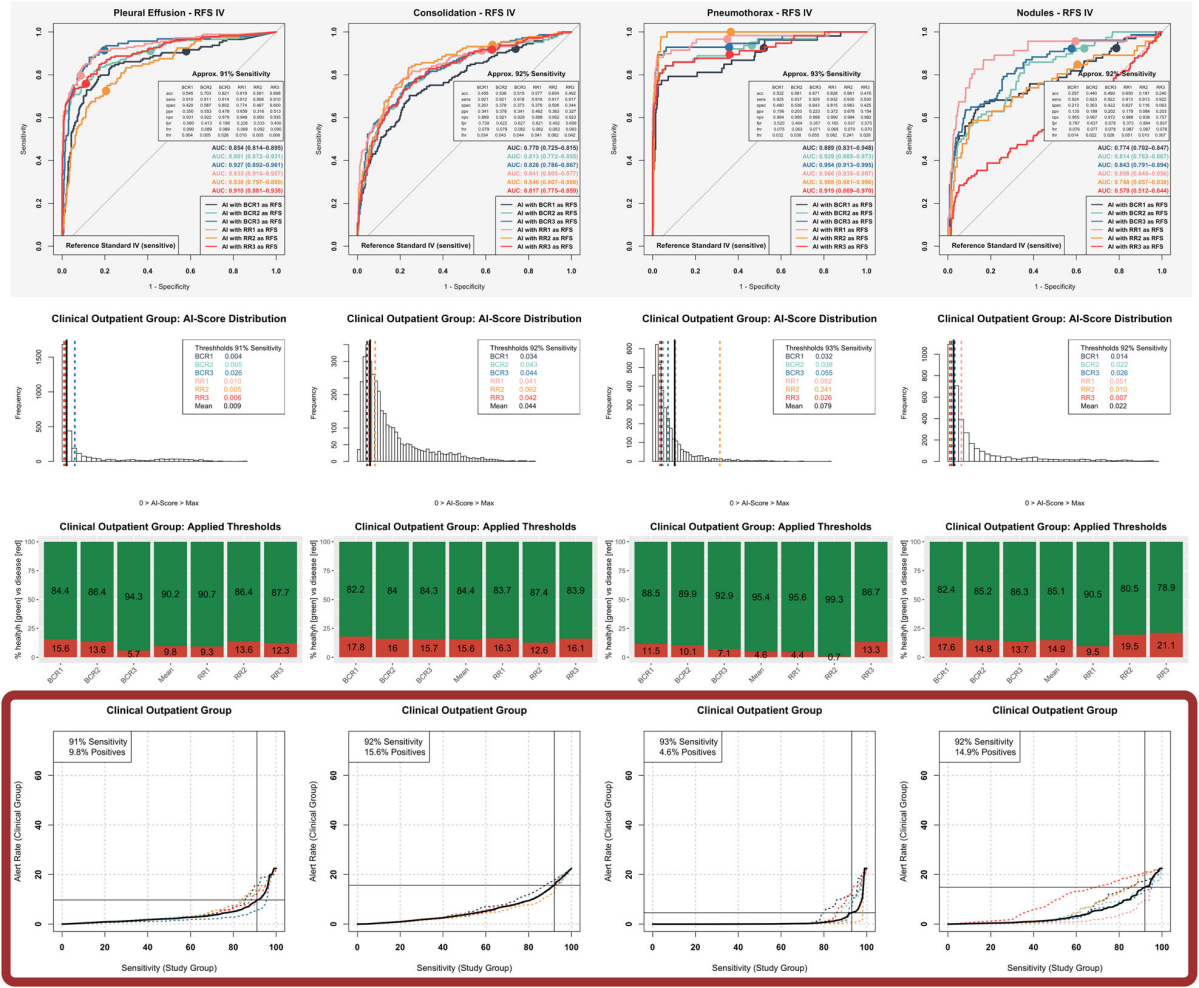
Optimization of AI algorithm thresholds based on the reference standard (RFS) IV study group and clinical outpatient group. Column-wise depiction of distinct pathologies, including pleural effusion, consolidations suspicious for pneumonia, pneumothorax and nodules (from left to right). Top row: ROC analysis of AI performance in the study group, separately referenced to the RFS IV of six different reference readers. Exemplarily illustrated diagnostic metrics refer to optimized thresholds (OPOTs). Second row: Histogram illustrating the distribution of AI scores in the clinical outpatient group, with highlighted optimized thresholds (OPOT) separately derived from individual reference readers and their common mean (black). Third row: Resulting percentages of images classified as positive (red) based on the optimized thresholds of the different reference readers and their collective mean (black). Bottom row (key information/framed in dark red): Relationship between achievable AI sensitivity in the study cohort and the resulting percentage of chest radiographs AI-classified as positive (alert rate) in the clinical outpatient group. The target sensitivity is derived from the mean curve (black) of the different reference readers (dotted and colored), where the slope first exceeds one. The target sensitivity and corresponding alert rate are depicted in the top-left corner. acc, Accuracy; BCR, Board-certified radiologist; fnr, False-negative rate; fpr, False-positive rate; npv, Negative predictive value; ppv, Positive predictive value; RR, Radiology resident; sens, Sensitivity; spec, Specificity; thr, Threshold

**Fig. 3:**
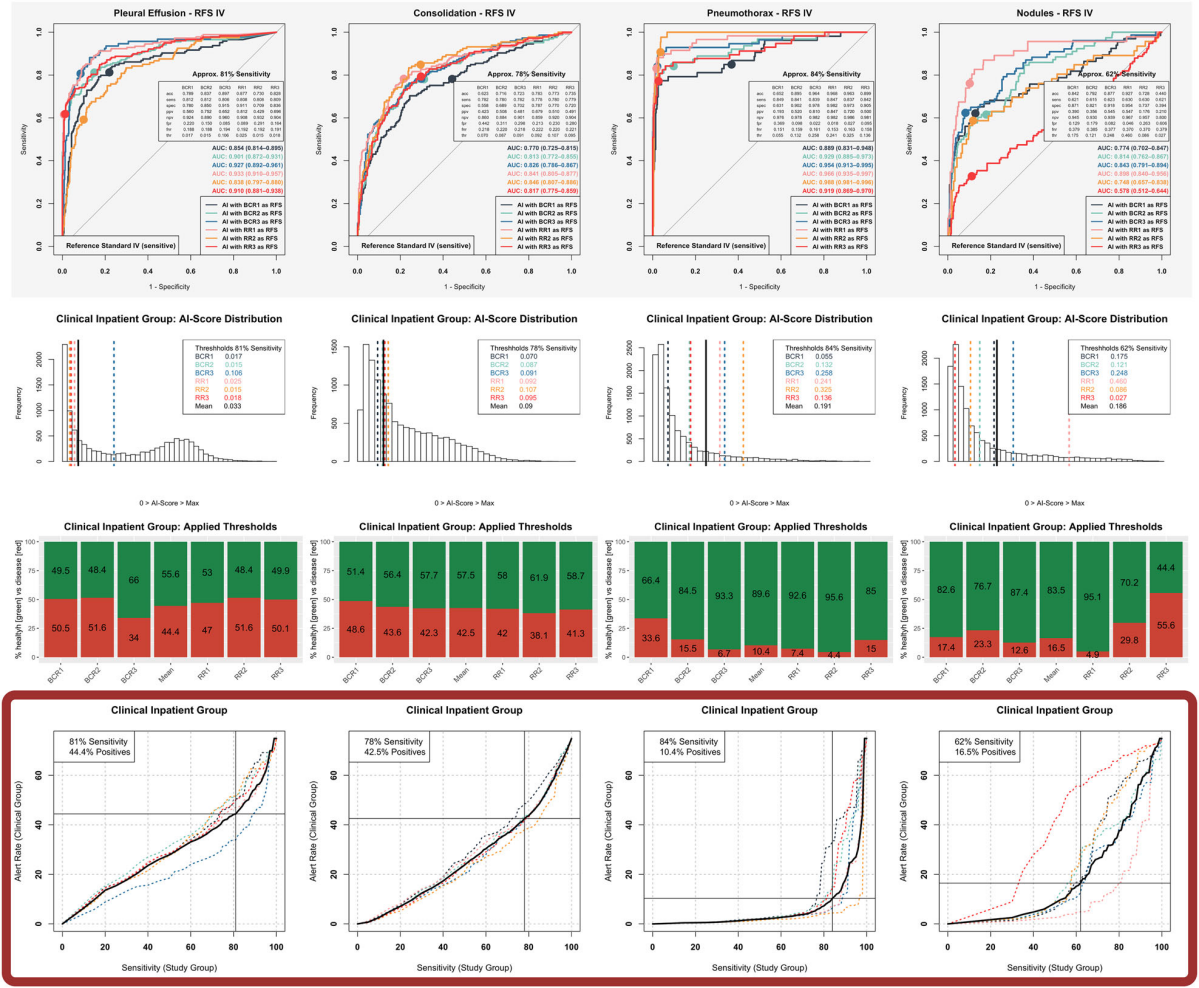
Optimization of artificial intelligence (AI) algorithm thresholds based on the reference standard (RFS) IV study group and clinical inpatient group. Column-wise depiction of distinct pathologies, including pleural effusion, consolidations suspicious for pneumonia, pneumothorax, and nodules (from left to right). Top row: receiver operating characteristic analysis of AI performance in the study group, separately referenced to the RFS IV of six different reference readers. Exemplarily illustrated diagnostic metrics refer to optimized thresholds (IPOT). Second row: histogram illustrating the distribution of AI scores in the clinical inpatient group, with highlighted optimized thresholds (IPOT) separately derived from individual reference readers and their common mean (black). Third row: percentages of images classified as positive (red) based on the optimized thresholds of the different reference readers and their collective mean (black). Bottom row (key information/framed in dark red): relationship between achievable AI sensitivity in the study cohort and the resulting percentage of chest radiographs AI-classified as positive (alert rate) in the clinical inpatient cohort. The target sensitivity is derived from the mean curve (black) of the different reference readers (dotted and colored), where the slope first exceeds one. The target sensitivity and corresponding alert rate in the clinical inpatient cohort are depicted in the top-left corner. acc, Accuracy; BCR, Board-certified radiologist; fnr, False-negative rate; fpr, False-positive rate; npv, Negative predictive value; ppv, Positive predictive value; RR, Radiology resident; sens, Sensitivity; spec, Specificity; thr, Threshold

**Fig. 4:**
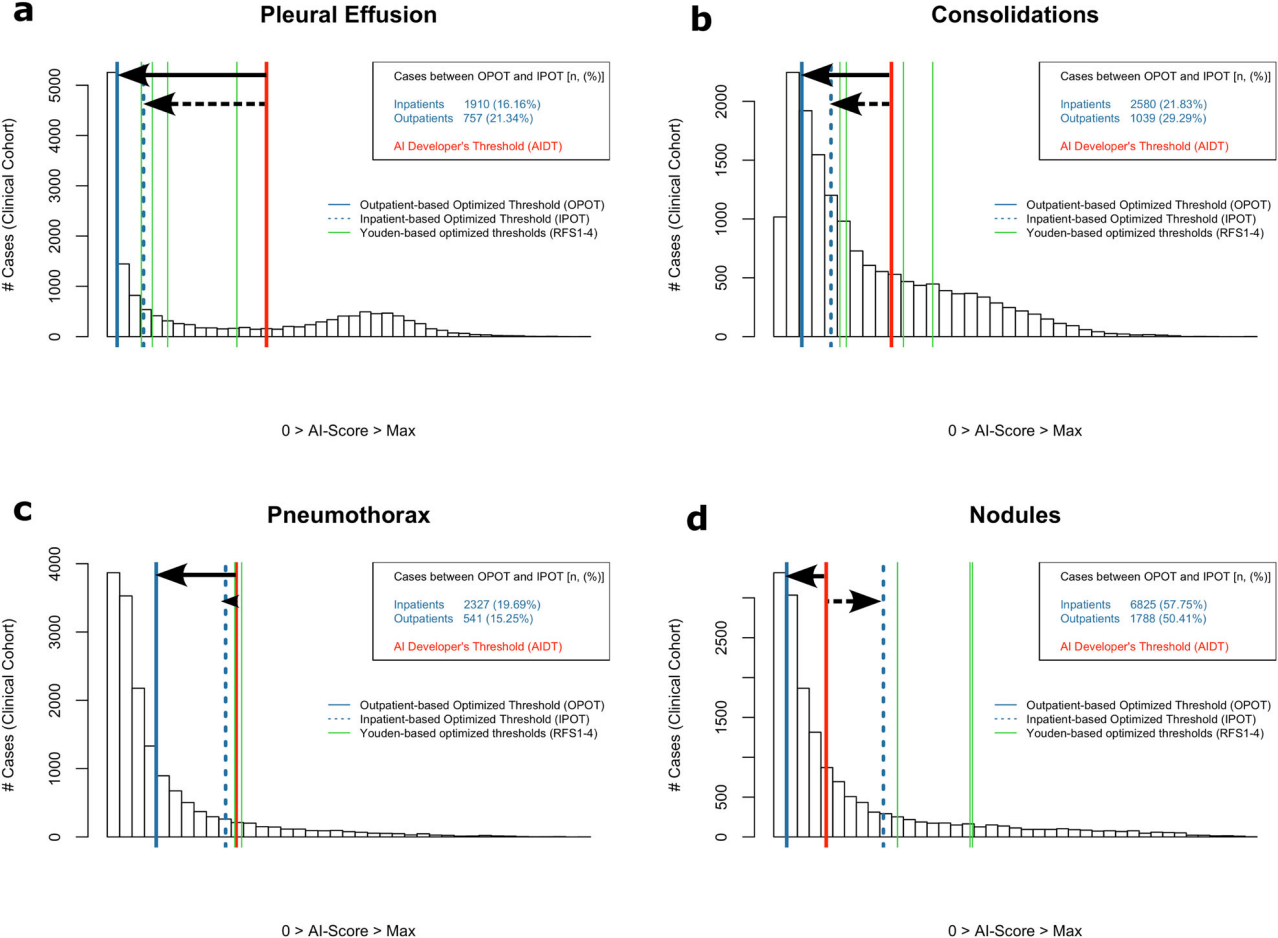
Comparative analysis of differentially optimized thresholds applied to the clinical cohort for (**a**) pleural effusion, (**b**) consolidations, (**c**) pneumothorax, and (**d**) nodules. The background histogram aggregates data from both inpatients and outpatients. Thresholds sensitized based on study group reference standard (RFS) IV are blueish depicted. The AI developer’s thresholds (AIDT) are shown in red. Black arrows indicate the threshold shift from the AIDT to those optimized for inpatients (IPOT, solid arrows) and for outpatients (OPOT, dashed arrows) within the AI score spectrum. Thresholds optimized based on Youden’s J statistic [31] based on study groups RFS I–IV (the more sensitive the RFS, the lower the cutoffs, from right to left) are highlighted in green (traditional threshold optimization method). The box on the right provides a breakdown of the total number and percentage of clinical inpatient and outpatient images falling between OPOT and IPOT. In scenarios where patient-subgroup-specific thresholds would be applied, these cases would be AI-categorized as positive or negative for disease, depending on their known inpatient or outpatient status

The six reference reader-specific ROC curves were systematically sampled based on various sensitivities and the underlying AI thresholds; these thresholds were applied to the clinical cohort, and the resulting proportions of positively rated CXRs (alert rates) were quantified. This approach elucidates the threshold-dependent relationship between achievable sensitivity in the pathology-enriched study group (prevalence-independent and supposed to be transferable) and the resulting alert rates in clinical routine. These relationships are graphically illustrated as a key baseline for subsequent threshold selection/optimization by slope analysis (Figs. 2, 3, bottom row). Diagnostic performances of AI thresholds differentially optimized based on clinical outpatients (outpatient group optimized threshold (OPOT)) and clinical inpatients (inpatient group optimized threshold (IPOT)) were compared with the default AI developer’s thresholds (AIDT) as well as with thresholds traditionally optimized according to Youden’s J criterion (Fig. 4). Threshold comparisons included resulting diagnostic metrics (*e.g*., sensitivity, specificity, accuracy) and the alert rates, which were quantified based on reference readings provided for 400 randomly selected clinical group cases (Tables 2, 3). Statistical calculations and graphical illustrations were conducted using the open-source programming language R [30]. Figure 1 presents a flowchart summarizing the analysis pipeline as described above.

**Table 2:** Diagnostic metrics of optimized thresholds and AI developer’s thresholds for the detection of pleural effusions and consolidations

Threshold optimized for			Outpatient group (OPOT)		Inpatient group (IPOT)		AI developer’s threshold (AIDT)	
Reference standard/algorithm versions			RFS IV		RFS IV		AI Algorithm (V10)	
Metrics in group			Outpatients	Inpatients	Outpatients	Inpatients	Outpatients	Inpatients
Pleural effusion	Threshold		**0.009**		**0.033**		**0.145**	
	Sensitivity		87.2% [77.7; 96.8]% ***p*** **< 0.001**	98.9% [96.8; 100.0]% ***p*** **< 0.001**	63.8% [50.1; 77.6]% *p* = 0.146	93.5% [88.6; 98.5]% ***p*** **= 0.002**	46.8% [32.5; 61.1]%	76.3% [67.7; 85.0]%
	Specificity		69.3% [62.0; 76.6]% ***p*** **< 0.001**	46.7% [37.3; 56.2]% ***p*** **< 0.001**	88.2% [83.1; 93.3]% ***p*** **= 0.002**	66.4% [57.4; 74.3]% ***p*** **< 0.001**	98.0% [95.8; 100.0]%	88.8% [82.8; 94.8]%
	Accuracy		73.5% [67.4; 79.6]% ***p*** **= 0.003**	71.0% [64.7; 77.3]% ***p*** **= 0.006**	82.5% [77.2; 87.8]% *p* = 0.410	79.0% [73.4; 84.6]% *p* = 0.372	86.0% [81.2; 90.9]%	83.0% [77.8; 88.2]%
	Positives (alert rate) in clinical cohort (*n*, %)	Outpatients	1,592 (44.0%)		816 (22.5%)		385 (10.6%)	
		Inpatients	8,957 (75.3%)		7,037 (59.1%)		4,836 (40.6%)	
		Overall cohort	10,713 (67.9%)		7,975 (50.5%)		5,303 (33.6%)	
Consolidation	Threshold		**0.044**		**0.090**		**0.185**	
	Sensitivity		97.9% [93.7; 100.0]% ***p*** **< 0.001**	96.5% [91.9; 100.0]% ***p*** **< 0.001**	80.9% [69.6; 92.1]% ***p*** **= 0.008**	82.8% [73.0; 92.5]% ***p*** **= 0.008**	53.2% [38.9; 67.5]%	58.6% [45.9; 71.3]%
	Specificity		29.4% [22.2; 36.6]% ***p*** **< 0.001**	33.8% [26.0; 41.6]% ***p*** **< 0.001**	68.0% [60.6; 75.4]% ***p*** **< 0.001**	58.5% [50.3; 66.6]% ***p*** **< 0.001**	86.9% [81.6; 92.3]%	85.2% [79.4; 91.0]%
	Accuracy		45.5% [38.6; 52.4]% ***p*** **< 0.001**	52.0% [45.1; 58.9]% ***p*** **< 0.001**	71.0% [64.7; 77.3]% *p* = 0.083	65.5% [58.9; 72.1]% ***p*** **= 0.011**	79.0% [73.4; 84.6]%	77.5% [71.7; 83.3]%
	Positives (alert rate) in clinical cohort (*n*, %)	Outpatients	2,530 (69.9%)		1,458 (40.3%)		657 (18.2%)	
		Inpatients	9,365 (78.7%)		6,759 (56.8%)		4,018 (33.8%)	
		Overall cohort	12,073 (76. %)		8,325 (52.7%)		4,731 (30.0%)	

Diagnostic metrics (sensitivity, specificity, accuracy) were calculated based on randomly selected chest x-ray cases (200 inpatient and outpatient) for a board-certified radiologist’s reference reading. 95% confidence intervals are shown in square brackets. The reported *p*-values refer to comparisons with the AIDT-related metrics. The thresholds used and statistically significant *p*-values are highlighted in bold. Additional diagnostic metrics are illustrated in the Supplementary tables

*IPOT* Inpatient group optimized threshold, *OPOT* Outpatient group optimized threshold

**Table 3:** Diagnostic metrics of optimized thresholds and AI developer’s thresholds for the detection of pneumothorax and nodules

Threshold optimized for			Outpatient group (OPOT)		Inpatient group (IPOT)		AI developer’s threshold (AIDT)	
Reference standard/algorithm versions			RFS IV		RFS IV		AI Algorithm (V10)	
Metrics in group			Outpatients	Inpatients	Outpatients	Inpatients	Outpatients	Inpatients
Pneumothorax	Threshold		**0.079**		**0.191**		**0.208**	
	Sensitivity		80.0% [44.9; 100.0]% *p* = 1	96.0% [88.3; 100.0]% *p* = 0.103	80.0% [44.9; 100.0]% *p* = 1	76.0% [59.3; 92.7]% *p* = 1	80.0% [44.9; 100.0]%	76.0% [59.3; 92.7]%
	Specificity		74.4% [68.2; 80.5]% ***p*** < **0.001**	71.4% [64.7; 78.1]% ***p*** < **0.001**	95.4% [92.4; 98.3]% *p* = 0.798	89.7% [85.2; 94.2]% *p* = 1	96.4% [93.8; 99.0]%	90.3% [85.9; 94.7]%
	Accuracy		74.5% [68.5; 80.5]% ***p*** < **0.001**	74.5% [68.7; 78.1]% ***p*** < **0.001**	95.0% [92.0; 98.0]% *p* = 0.809	88.0% [83.5; 92.5]% *p* = 1	96.0% [93.3; 98.7]%	88.5% [84.1; 92.9]%
	Positives (alert rate) in clinical cohort (*n*, %)	Outpatients	740 (20.4%)		191 (5.3%)		167 (4.6%)	
		Inpatients	3,995 (33.5%)		1,653 (13.9%)		1,479 (12.4%)	
		Overall cohort	4,813 (30.5%)		1,875 (11.9%)		1,673 (10.6%)	
Nodules	Threshold		**0.022**		**0.186**		**0.089**	
	Sensitivity		93.8% [81.9; 100.0]% *p* = 0.593	100.0%, [100.0; 100.0]% *p* = 1	68.8% [46.0; 91.5]% *p* = 0.683	90.9% [73.9; 100.0]% *p* = 1	81.3% [62.1; 100.0]%	90.9% [73.9; 100.0]%
	Specificity		32.6% [25.8; 39.4]% ***p*** < **0.001**	29.6% [23.1; 36.1]% ***p*** < **0.001**	87.5% [82.7; 92.3]% ***p*** = **0.019**	82.0% [76.5; 87.5]% ***p*** = **0.003**	77.7% [71.7; 83.7]%	68.3% [61.6; 74.9]%
	Accuracy		37.5% [30.8; 44.2]% ***p*** < **0.001**	33.5% [27.0; 40.0]% ***p*** < **0.001**	86.0% [81.2; 90.8]% *p* = 0.051	82.5% [77.2; 87.8]% ***p*** = **0.003**	78.0% [72.3; 83.7]%	69.5% [63.1; 75.9]%
	Positives (alert rate) in clinical cohort (*n*, %)	Outpatients	2,366 (65.4%)		545 (15.1%)		973 (26.9%)	
		Inpatients	9,477 (79.6%)		2,613 (22.0%)		4,607 (38.7%)	
		Overall cohort	12,033 (76.2%)		3,203 (20.3%)		5,666 (35.9%)	

Diagnostic metrics (sensitivity, specificity, accuracy) were calculated based on randomly selected chest x-ray cases (200 inpatient and outpatient) for a board-certified radiologist’s reference reading. 95% confidence intervals are shown in square brackets. The reported *p*-values refer to comparisons with the AIDT-related metrics. The thresholds used and statistically significant *p*-values are highlighted in bold. Additional diagnostic metrics are illustrated in the Supplementary tables

*IPOT* Inpatient group optimized threshold, *OPOT* Outpatient group optimized threshold

## Results

The process of pathology-specific AI threshold optimization, based on both the study and clinical subgroup cohorts, has been established as an automated pipeline with graphical results illustration, as shown in Fig. 2 (outpatient subgroup) and Fig. 3 (inpatient subgroup) for the underlying RFS IV (equivalent results based on the less sensitive RFSs I-III are provided as Supplementary material). The bottom row subgraphs (Figs. 2, 3, highlighted by framing) represent a key interim result of the study approach, illustrating the relationship between achievable AI sensitivity for pathology detection and the corresponding alert rate in clinical practice, both linked by individually selectable AI thresholds. These subgraphs formed the basis for OPOT/IPOT calculation via slope analysis, based on the study-specific definition introduced earlier.

**Fig. 5:**
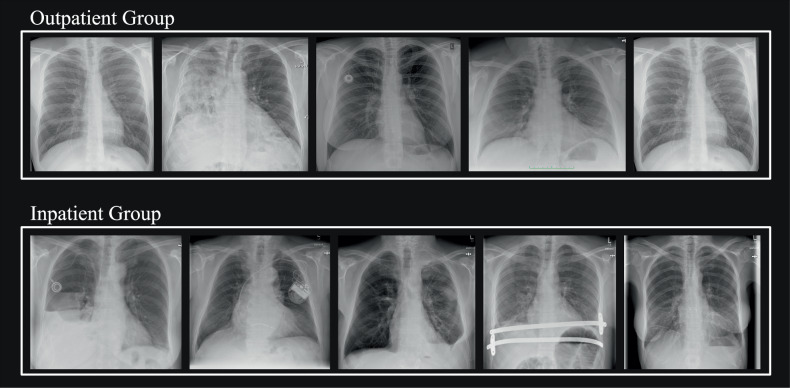
Representative clinical cohort cases. The figure displays five randomly selected case examples from both the outpatient and inpatient groups of the clinical cohort. The series suggests that the outpatient group generally consists of less complex cases, with fewer co-occurring pathologies and a higher proportion of “unremarkable” cases. In contrast, the inpatient group typically shows greater case complexity. For example, the series includes postoperative images and a higher number of cases involving patients with long-term implanted devices (*e.g*., port catheters or pacemakers)

Study-specific OPOTs/IPOTs are depicted alongside the AI score distribution histogram, compared with AIDTs and thresholds traditionally optimized using Youden’s J statistics [31] (Fig. 4). Diagnostic metrics resulting from the different thresholds are comparably summarized in Tables 2 and 3, with additional visual details available in Figs. 2–4. Figure 5 provides examples of five randomly selected cases from the clinical outpatient and inpatient cohorts, illustrating the potential difference of pathology complexity in the different cohorts. Additional pathology-specific contents, also including other RFSs can be found in the Supplementary Tables S1–S4 and Figs. S1–S4.

### Pleural effusion detection

The OTs (IPOTs/OPOTs) as well as thresholds optimized according to Youden’s J criterion were noticeably lower compared to the AIDT for in- as well as for outpatient analysis (Fig. 4a). Regarding OT-associated metrics as a boundary consideration of maximal sensitization: sensitivity in the outpatient subgroup significantly (*p* < 0.001) increased from 46.8% (AIDT) to 87.2% (OPOT), nevertheless accompanied by an increase in the alert rate from 10.6% (AIDT) to 44.0% (OPOT), exceeding the estimated clinical outpatient group prevalence of 23.5% (Tables 1, 2). Similarly, for inpatients, sensitivity significantly (*p* = 0.002) rose from 76.3% (AIDT) to 93.5% (IPOT), associated with an increase in the alert rate from 40.6% (AIDT) to 59.1% (IPOT), exceeding the estimated clinical inpatient prevalence of 46.5% (Tables 1, 2). The tradeoff (AIDT-related metrics *versus* OT-related metrics) in accuracy was statistically significant for outpatients (accuracy drop from 86.0% to 73.5%, *p* = 0.003), while for inpatients, the change in accuracy was not statistically significant (from 83.0% to 79.0%, *p* = 0.372) (Table 2). As an alternative to the quantified OT thresholds, the required level of AIDT sensitization can also be estimated within an intermediate range, as indicated by the optimization subgraphs (Figs. 2, 3, bottom row, left).

### Consolidation/pneumonia detection

The OTs (IPOTs/OPOTs) as well as thresholds optimized according to Youden’s J criterion based on the more sensitive RFSs III/IV were lower compared to the AIDT for in- as well as for outpatient analysis (Fig. 4b). Regarding OT-associated metrics as a boundary consideration of maximal sensitization: Sensitivity in the outpatient subgroup significantly (*p* < 0.001) increased from 53.2% (AIDT) to 97.9% (OPOT), though accompanied by a rise in alert rate up to 69.9% (Table 2), far exceeding the estimated prevalence of 23.5% (Table 1). Similarly, for inpatients, sensitivity significantly (*p* = 0.008) increased from 58.6% (AIDT) to 82.8% (IPOT), again associated with an increased alert rate up to 56.8%, noticeably exceeding the estimated prevalence of 29.0% (Tables 1, 2). The tradeoff (AIDT-related metrics *versus* OT-related metrics) in accuracy was statistically significant again for outpatient analysis (Table 2). Also here, the required level of AIDT sensitization can be estimated within an intermediate range as an alternative to the quantified OT thresholds, *e.g*., based on the provided optimization subgraphs (Figs. 2, 3, bottom row).

### Pneumothorax detection

AIDTs matched well with the IPOT as well as with the Youden’s J thresholds; only the OPOT was notably lower than the AIDT (Fig. 4c). Unfortunately, a possibly beneficial effect of AIDT lowering for outpatient analysis could not be further quantified for statistical reasons: The low pneumothorax prevalence of estimated 2.5% in the clinical outpatient group (Table 1) limited the quantification of diagnostic metrics, as the limited number of clinical outpatient group cases selected for radiologists’ reference reading (Table 3) was insufficient to demonstrate a sensitivity gain by threshold lowering (Table 3).

### Nodule detection

A different effect was observed. Both the calculated IPOT and Youden’s J threshold exceed the AIDT, with only the OPOT falling below (Fig. 4d). For the inpatient subgroup, the use of IPOT instead of AIDT maintained high sensitivity at 90.9% while significantly improving accuracy (from 69.5% to 82.5%, *p* = 0.003) and reducing alert rates from 38.7% to 22.0% (Table 3), which outlines a relevant desensitization potential. Contrarily, in outpatients, while OPOT non-significantly increased sensitivity from 81.3% to 93.8% (*p* = 0.593), this was offset by a significant drop in accuracy (from 78.0% to 37.5%, *p* < 0.001) and a sharp rise in alert rate up to 65.4%, far exceeding the prevalence of 5.5% (Tables 1, 3).

## Discussion

The presented study proposes an innovative AI algorithm threshold optimization strategy that extends traditional methods, such as Youden’s J method, commonly applied/limited to pathology-enriched datasets, apart from the medical users’ clinical routine. Unlike these conventional approaches, the proposed method enables a direct correlation between sensitivity and AI alert rate—naturally accounting for false positives—thereby aligning optimization more closely with the clinical reality and the practical burden of alerts. The presented approach additionally enables the consideration of large-scale real-world imaging datasets, the differential incorporation of clinical subgroup considerations, to individually refine threshold optimization and to better address specific needs of medical expert users or patient subgroups. This approach was exemplarily demonstrated to uncover threshold optimization potentials of a pre-commercial, state-of-the-art AI system for chest radiography analysis, here with inpatient and outpatient subgroups separately considered. The study results demonstrate the effectiveness of the proposed strategy in contributing to AI threshold optimization within a clinically representative setting, specifically by drawing attention to thresholds notably deviating from the AI vendor’s predefined thresholds. Furthermore, the findings underscore the potential value of individually adjustable thresholds tailored to specific patient subgroups, as exemplified by the differentially optimized thresholds for inpatients and outpatients.

### Potential clinical applications

The results of this study have several potential clinical applications, particularly in tailoring diagnostic strategies for specific patient subgroups. Variations in subgroup-specific requirements for algorithmic sensitivity and specificity may warrant individual threshold optimization based on clinical context and patient characteristics. For example, sensitized thresholds for infection-related pathologies could be particularly valuable for hospitalized patients, where early detection of high-prevalence nosocomial pneumonia and prompt initiation of antibiotic therapy might improve patient outcomes [32, 33]. Additionally, adjustable thresholds could be useful in settings involving co-occurring pathologies; for instance, in cases of unilateral pleural effusion, sensitized AI thresholds might be appropriate for detecting co-occurring malignancy or infection. In low-prevalence settings, such as ambulatory chest x-rays in the emergency department, sensitized thresholds could be used to optimize rule-out triage for AI-negative examinations, ensuring accurate identification of cases requiring no further follow-up. Furthermore, recent studies have shown that threshold optimization can enhance the diagnostic accuracy of AI algorithms [34, 35] and even expand their application to new areas [36]. For instance, a recent study by Shin et al demonstrated that threshold optimization improved the diagnostic performance of an algorithm trained on adult CXRs when applied to pediatric CXRs [36]. This example highlights how threshold optimization can be beneficial not only within specific subgroups but also when extending applications to distinct patient populations, such as pediatric cases.

### AI perception and threshold optimization

In most studies conducted for the external validation of CXR interpreting AI algorithms, algorithm performance is quantified by prevalence-independent parameters (such as sensitivity and specificity), allowing for the investigation of low-prevalence pathologies within necessarily enriched cohorts [3, 14–18]. However, based on our experience, the individual perception of diagnostic AI algorithms in clinical practice is at least equally influenced by prevalence-dependent metrics, such as predictive values or accuracy. Particularly, for low-prevalence pathologies, it is not feasible to perform validation and threshold optimization on large-scale clinical routine datasets, as this would require extensive expert readings. Alternatively, without the need for an unmanageable volume of expert readings, the distribution of the resulting AI scores can be used in threshold optimization strategies. Such an approach allows for a balance between achieving high sensitivity (not depending on prevalence, which can be derived from synthetic datasets) and maintaining acceptable AI alert rates (derived from AI score distribution in clinical practice). This innovative approach deviates from traditional optimization strategies, such as those based on Youden’s J index, typically calculated using ROC analyses of synthetic datasets.

### Proposed optimization pipeline with strengths and limitations

The threshold optimization pipeline proposed by this study represents a structured methodology that enables the identification of algorithm threshold optimization potential, *e.g*., as compared with the AIDTs, and enables the incorporation of different clinical routine datasets, including varying patient subgroups and radiology professional habits. In contrast to classical thresholding, *e.g*., according to Youden’s J statistics on synthetic datasets, the methodology presented here additionally considers prevalence-dependent effects in clinical and subgroup-specific routine.

The strength of the proposed pipeline lies in its versatility, enabling the simultaneous calculation of multiple parameters using both a pathology-enriched study cohort (563 cases) with a strong reference standard from six expert readers and a clinically representative 1-year cohort (15,786 cases). The approach first calculates ROC curves for individual reference readers, addressing inter-reader variability. Key results include the quantification of threshold-dependent relationships between expected sensitivities (derived from the study cohort) and associated alert rates in clinical practice, which are used to extract optimized thresholds for specific subgroups through slope analysis. Interestingly, up to here, the entire automated thresholding pipeline can incorporate alternative clinical routine datasets from other institutions, including different clinical subgroup definitions, without the need for additional expert readings. This ensures the reproducibility of the strategy across various clinical contexts and for different CXR analysis AI models, by illustrating the relationship between pathology detection sensitivity and subgroup-specific AI alert rates (see Figs. 2, 3, bottom rows), ultimately enabling individualized threshold selection. This reproducibility and transferability to different clinical subgroup settings, as well as the resulting comparability with the AI vendor’s default thresholds, is the key strength of the proposed thresholding strategy. Taken together, this approach provides valuable insights not only into the algorithm itself but also into its relevance for deployment in clinical routine subgroups and, more specifically, its potential for subgroup-specific adaptation through threshold optimization.

Limitations of the proposed thresholding strategy include the reliance on a single-center pathology-enriched dataset composed exclusively of posteroanterior views—potentially excluding patients unable to undergo upright imaging—and the fact that all six reference readers presumably have comparable training. At present, our clinical cohort cannot be supplemented or replaced by larger publicly available datasets to achieve a multicenter design, as—based on our current knowledge—comparable subgroup classifications (specifically inpatients *versus* outpatients) are not available in existing datasets. The proposed optimization pipeline operates on binary outputs and does not account for potential AI uncertainty. Furthermore, the final strategy step of comparing threshold-related metrics in the clinical routine subgroup requires at least a small sample reading of the incorporated clinical subgroup datasets, which in this study was based on a limited number of 400 sample readings, thus limiting statistical power, particularly for low-prevalence pathologies (*e.g*., pneumothorax in the outpatient group). Moreover, differences in CXR quality or the extent of pathology between the pathology-enriched dataset and the incorporated clinical routine datasets might introduce potential bias, as the ROC analysis of the enriched dataset is used to predict sensitivity in clinical practice. Also, there is a small overlap of cases between the study and clinical cohorts (120 cases = 0.8%), which is even smaller in the subset with targeted reading (6 cases; 0.04%), and is therefore considered negligible for the purposes of this analysis. It should be noted that when working with Conformité Européenne (CE)-cleared products—particularly in regulated sectors such as medical devices or diagnostics—it is essential to ensure full compliance with applicable European regulations.

Future studies should be expanded to include a wider range of pathologies, should incorporate additional clinical subgroups from multiple clinical facilities (where appropriate, technically normalized [37, 38]), and should, in this way, explore the potential of further threshold individualization, *e.g*., depending on suspicious pathology co-occurrences. To support such efforts and ensure methodological generalizability, the proposed approach can be readily applied to other AI models, clinical cohorts, or subgroup definitions, enabling alternative, tailored threshold selection while transparently accounting for the resulting sensitivities and alert rates across diverse subpopulations.

In conclusion, we provide proof-of-concept evidence that individualized AI threshold optimization can offer benefits for distinct clinical subgroups, particularly by incorporating subgroup-specific factors (*e.g*., AI alert rates influenced by disease prevalence) into innovative thresholding strategies. Moreover, provided that regulatory frameworks allow, user-adapted adjustment of threshold settings may also be a feasible and promising approach to further enhance clinical applicability, performance and perception of AI-based diagnostic tools.

## Supplementary information

**Additional file 1:** **Fig. S1.** Complete Illustration of Threshold Optimization for the Pathology Pleural Effusion. Column-wise illustration for the analysis based on the underlying increasingly sensitive study cohort reference standards I-IV (from left to right). The upper four rows illustrate the threshold optimization for the clinical outpatient group. The lower four rows illustrate the threshold optimization for the clinical inpatient group. The individual subfigures correspond to these ones illustrated in Figs. 1 and 2 in the main part of the manuscript, please compare with the associated captions. **Fig. S2.** Complete Illustration of Threshold Optimization for the Pathology Consolidations Suspicious for Pneumonia. Column-wise illustration for the analysis based on the underlying increasingly sensitive study cohort reference standards I-IV (from left to right). The upper four rows illustrate the threshold optimization for the clinical outpatient group. The lower four rows illustrate the threshold optimization for the clinical inpatient group. The individual subfigures correspond to these ones illustrated in Figs. 1 and 2 in the main part of the manuscript, please compare with the associated captions. **Fig. S3.** Complete Illustration of Threshold Optimization for the Pathology Pneumothorax. Column-wise illustration for the analysis based on the underlying increasingly sensitive study cohort reference standards I-IV (from left to right). The upper four rows illustrate the threshold optimization for the clinical outpatient group. The lower four rows illustrate the threshold optimization for the clinical inpatient group. The individual subfigures correspond to these ones illustrated in Figs. 1 and 2 in the main part of the manuscript, please compare with the associated captions. **Fig. S4.** Complete Illustration of Threshold Optimization for the Pathology Suspicious Lung Nodules. Column-wise illustration for the analysis based on the underlying increasingly sensitive study cohort reference standards I-IV (from left to right). The upper four rows illustrate the threshold optimization for the clinical outpatient group. The lower four rows illustrate the threshold optimization for the clinical inpatient group. The individual subfigures correspond to these ones illustrated in Figs. 1 and 2 in the main part of the manuscript, please compare with the associated captions. **Table S1.** Estimated Metrics in Clinical Cohort with Optimized / AI Developer’s Threshold in Pathology Pleural Effusion. Given metrics are derived from expected prevalence according to results of a random sample reading of 200 cases respectively from the inpatient and outpatient group (see Table 1). 95% confidence intervals are shown in square brackets []. The reported *p*-values refer to comparisons of differences with the AI Developer Thresholds. The thresholds used and statistically significant *p*-values are highlighted in bold. **Table S2.** Estimated Metrics in Clinical Cohort with Optimized / AI Developer’s Threshold in Pathology Consolidation Suspicious for Pneumonia. Given metrics are derived from expected prevalence according to results of a random sample reading of 200 cases respectively from the inpatient and outpatient group (see Table 1). 95% confidence intervals are shown in square brackets []. The reported p-values refer to comparisons of differences with the AI Developer Thresholds. The thresholds used and statistically significant p-values are highlighted in bold. **Table S3.** Estimated Metrics in Clinical Cohort with Optimized / AI Developer’s Threshold in Pathology Pneumothorax. Given metrics are derived from expected prevalence according to results of a random sample reading of 200 cases respectively from the inpatient and outpatient group (see Table 1). 95% confidence intervals are shown in square brackets []. The reported p-values refer to comparisons of differences with the AI Developer Thresholds. The thresholds used and statistically significant p-values are highlighted in bold. Values highlighted with a gray background are subject to low statistical power due to low prevalence in the outpatient sample reading. **Table S4.** Estimated Metrics in Clinical Cohort with Optimized / AI Developer’s Threshold in Pathology Nodules. Given metrics are derived from expected prevalence according to results of a random sample reading of 200 cases respectively from the inpatient and outpatient group (see Table 1). 95% confidence intervals are shown in square brackets []. The reported p-values refer to comparisons of differences with the AI Developer Thresholds. The thresholds used and statistically significant *p*-values are highlighted in bold.

## Data Availability

Chest radiography images used in the pipeline are subject to local data protection regulations and cannot be publicly shared. Portions of the R scripts from this study are available upon reasonable request to the corresponding author. The scripts exclusively employ publicly available, pre-existing R packages without any modifications to their source code. Other chest radiography interpreting algorithms can be tested within the existing pipeline if provided to us.

## Abbreviations

AI: Artificial intelligence
AIDT: AI developer’s threshold
BCRs: Board-certified radiologists
CXR: Chest x-ray radiography
IPOT: Inpatient group optimized threshold
OPOT: Outpatient group optimized threshold
OT: Optimized threshold
RFS: Reference standard
ROC: Receiver operating characteristic
