# Supplementary material for: Threshold optimization in AI chest radiography analysis: integrating real-world data and clinical subgroups
Source: Eur Radiol Exp. 2025 Sep 22;9:95. doi: 10.1186/s41747-025-00632-8 (PMC12454861; doi:10.1186/s41747-025-00632-8)
Supplement: Supplementary file 1 — Additional file 1: Fig. S1. Complete Illustration of Threshold Optimization for the Pathology Pleural Effusion. Column-wise illustration for the analysis based on the underlying increasingly sensitive study cohort reference standards I-IV (from left to right). The upper four rows illustrate the threshold optimization for the clinical outpatient group. The lower four rows illustrate the threshold optimization for the clinical inpatient group. The individual subfigures correspond to these ones illustrated in Figs. 1 and 2 in the main part of the manuscript, please compare with the associated captions. Fig. S2. Complete Illustration of Threshold Optimization for the Pathology Consolidations Suspicious for Pneumonia. Column-wise illustration for the analysis based on the underlying increasingly sensitive study cohort reference standards I-IV (from left to right). The upper four rows illustrate the threshold optimization for the clinical outpatient group. The lower four rows illustrate the threshold optimization for the clinical inpatient group. The individual subfigures correspond to these ones illustrated in Figs. 1 and 2 in the main part of the manuscript, please compare with the associated captions. Fig. S3. Complete Illustration of Threshold Optimization for the Pathology Pneumothorax. Column-wise illustration for the analysis based on the underlying increasingly sensitive study cohort reference standards I-IV (from left to right). The upper four rows illustrate the threshold optimization for the clinical outpatient group. The lower four rows illustrate the threshold optimization for the clinical inpatient group. The individual subfigures correspond to these ones illustrated in Figs. 1 and 2 in the main part of the manuscript, please compare with the associated captions. Fig. S4. Complete Illustration of Threshold Optimization for the Pathology Suspicious Lung Nodules. Column-wise illustration for the analysis based on the underlying increasingly sensitive study coh [file 41747_2025_632_MOESM1_ESM.pdf]

ONLINE SUPPLEMENT – Figures

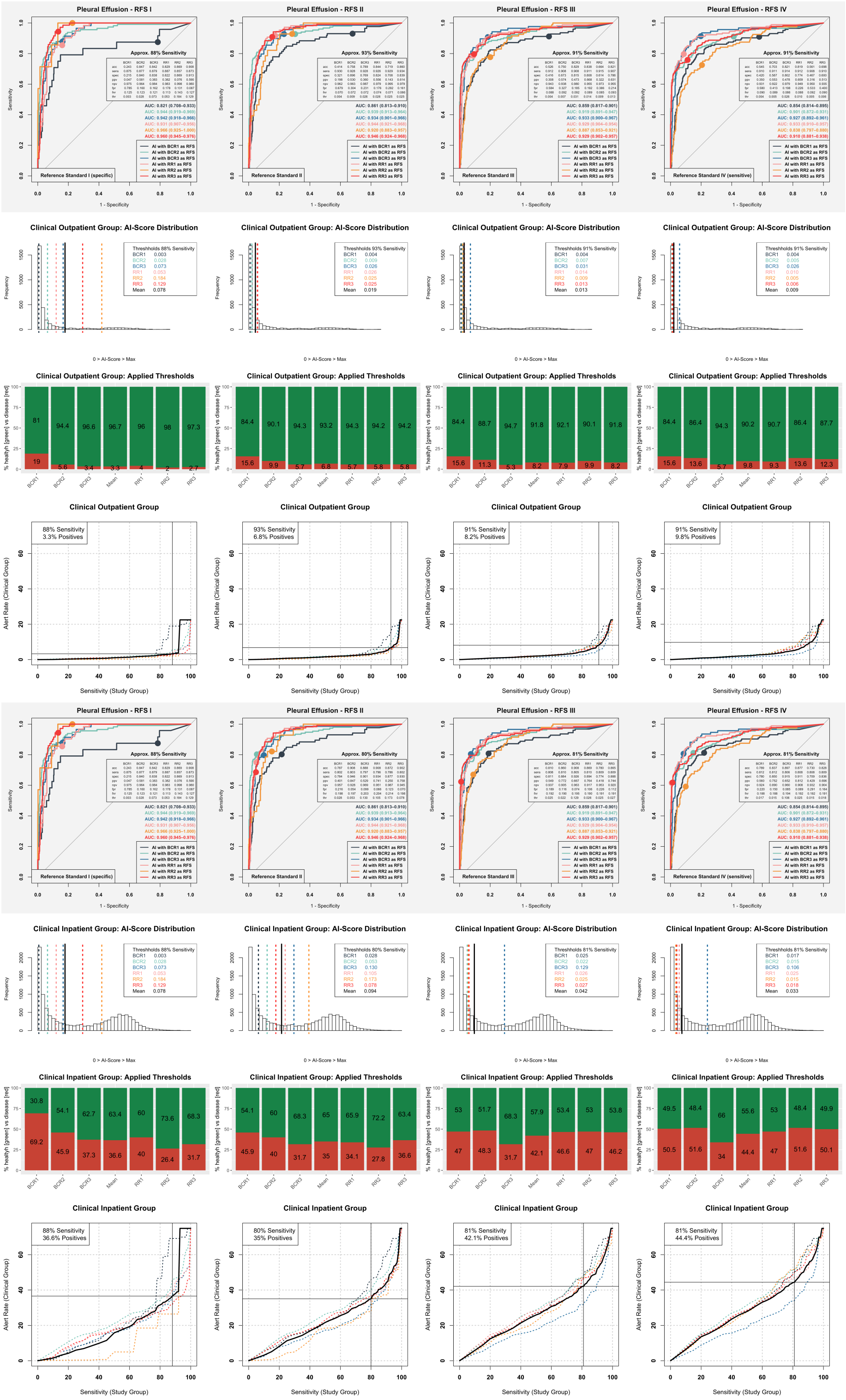

Fig. S1 - Complete Illustration of Threshold Optimization for the Pathology Pleural Effusion. Column-wise illustration for the analysis based on the underlying increasingly sensitive study cohort reference standards I-IV (from left to right). The upper four rows illustrate the threshold optimization for the clinical outpatient group. The lower four rows illustrate the threshold optimization for the clinical inpatient group. The individual subfigures correspond to these ones illustrated in Figs. 1 and 2 in the main part of the manuscript, please compare with the associated captions.

Further abbreviations: acc - accuracy, BCR – board-certified radiologist, fnr - false-negative rate, fpr - false-positive rate, npv - negative predictive value, ppv - positive predictive value, RR – radiology resident, sens - sensitivity, spec – specificity, thr – threshold

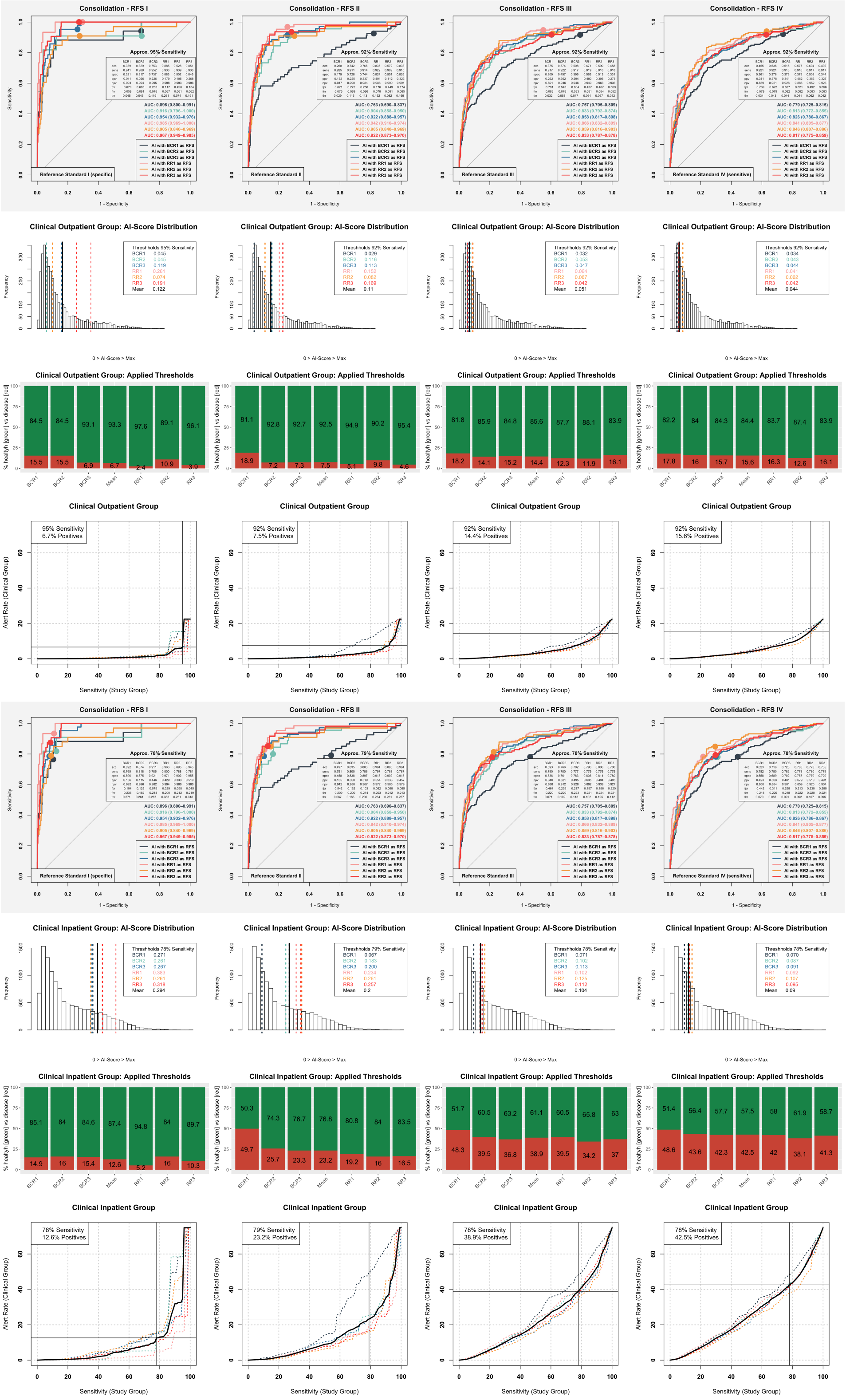

Fig. S2 - Complete Illustration of Threshold Optimization for the Pathology Consolidations Suspicious for Pneumonia. Column-wise illustration for the analysis based on the underlying increasingly sensitive study cohort reference standards I-IV (from left to right). The upper four rows illustrate the threshold optimization for the clinical outpatient group. The lower four rows illustrate the threshold optimization for the clinical inpatient group. The individual subfigures correspond to these ones illustrated in Figs. 1 and 2 in the main part of the manuscript, please compare with the associated captions. Further abbreviations: acc - accuracy, BCR – board-certified radiologist, fnr - false-negative rate, fpr - false-positive rate, npv - negative predictive value, ppv - positive predictive value, RR – radiology resident, sens - sensitivity, spec – specificity, thr – threshol

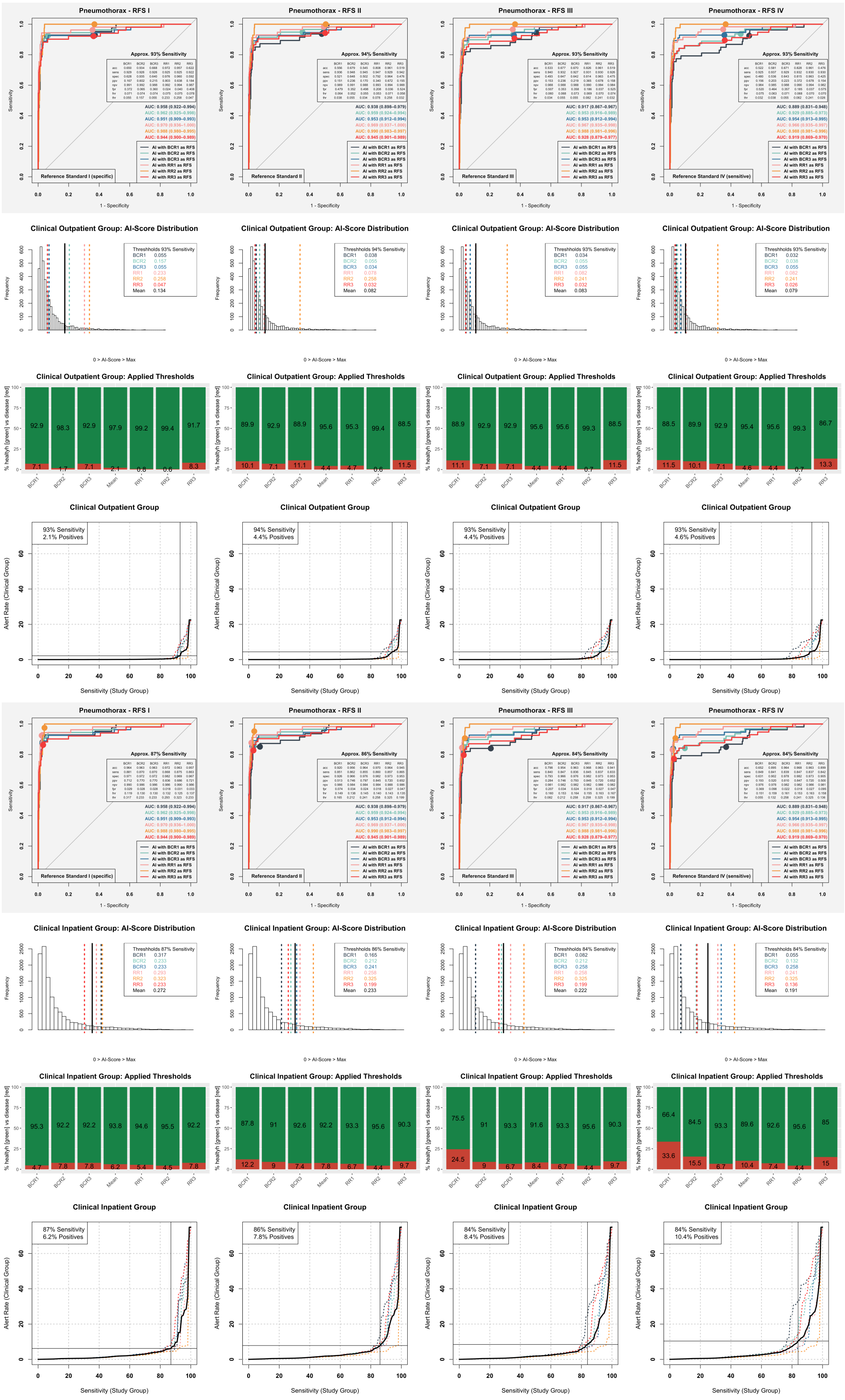

Fig. S3 - Complete Illustration of Threshold Optimization for the Pathology Pneumothorax. Column-wise illustration for the analysis based on the underlying increasingly sensitive study cohort reference standards I-IV (from left to right). The upper four rows illustrate the threshold optimization for the clinical outpatient group. The lower four rows illustrate the threshold optimization for the clinical inpatient group. The individual subfigures correspond to these ones illustrated in Figs. 1 and 2 in the main part of the manuscript, please compare with the associated captions. Further abbreviations: acc - accuracy, BCR – board-certified radiologist, fnr - false-negative rate, fpr - false-positive rate, npv - negative predictive value, ppv - positive predictive value, RR – radiology resident, sens - sensitivity, spec – specificity, thr – threshold

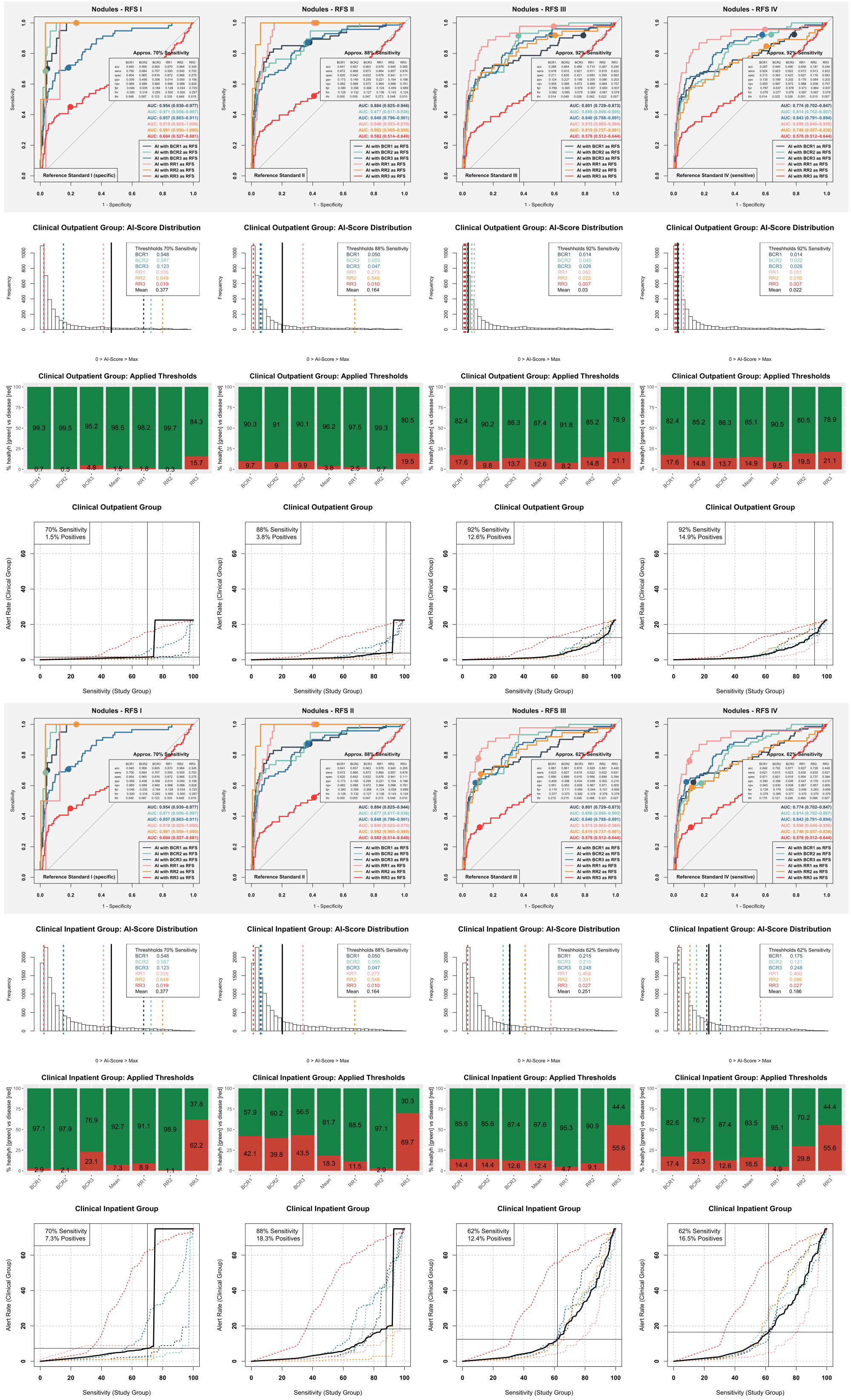

Fig. S4 - Complete Illustration of Threshold Optimization for the Pathology Suspicious Lung Nodules. Column-wise illustration for the analysis based on the underlying increasingly sensitive study cohort reference standards I-IV (from left to right). The upper four rows illustrate the threshold optimization for the clinical outpatient group. The lower four rows illustrate the threshold optimization for the clinical inpatient group. The individual subfigures correspond to these ones illustrated in Figs. 1 and 2 in the main part of the manuscript, please compare with the associated captions. Further abbreviations: acc - accuracy, BCR – board-certified radiologist, fnr - false-negative rate, fpr - false-positive rate, npv - negative predictive value, ppv - positive predictive value, RR – radiology resident, sens - sensitivity, spec – specificity, thr – threshold

ONLINE SUPPLEMENT – Tables

**Reference standard / algorithm versions**	**RFS III**				**RFS IV**				**algorithm version 10**	
**Threshold optimized for**	**Outpatient group<br>(OPOT)**		**Inpatient group<br>(IPOT)**		**Outpatient group<br>(OPOT)**		**Inpatient group<br>(IPOT)**		**AI developer's<br>threshold (AIDT)**	
**Metrics in group**	**outpatients**	**inpatients**	**outpatients**	**inpatients**	**outpatients**	**inpatients**	**outpatients**	**inpatients**	**outpatients**	**inpatients**
**Threshold**	**0.013**		**0.042**		**0.009**		**0.033**		**0.145**	
**Sensitivity**	85.1%<br>[74.9 ; 95.3]%<br>***p < 0.001***	96.8%<br>[93.2 ; 100.0]%<br>***p < 0.001***	61.7%<br>[47.8 ; 75.6]%<br>*p = 0.214*	92.5%<br>[87.1 ; 97.8]%<br>***p = 0.005***	87.2%<br>[77.7 ; 96.8]%<br>***p < 0.001***	98.9%<br>[96.8 ; 100.0]%<br>***p < 0.001***	63.8%<br>[50.1 ; 77.6]%<br>*p = 0.146*	93.5%<br>[88.6 ; 98.5]%<br>***p = 0.002***	46.8%<br>[32.5 ; 61.1]%	76.3%<br>[67.7 ; 85.0]%
**Specificity**	77.8%<br>[71.2 ; 84.4]%<br>***p < 0.001***	49.5%<br>[40.1 ; 59.0]%<br>***p < 0.001***	90.8%<br>[86.3 ; 95.4]%<br>***p = 0.013***	72.9%<br>[64.5 ; 81.3]%<br>***p = 0.005***	69.3%<br>[62.0 ; 76.6]%<br>***p < 0.001***	46.7%<br>[37.3 ; 56.2]%<br>***p < 0.001***	88.2%<br>[83.1 ; 93.3]%<br>***p = 0.002***	66.4%<br>[57.4 ; 74.3]%<br>***p < 0.001***	98.0%<br>[95.8 ; 100.0]%	88.8%<br>[82.8 ; 94.8]%
**Accuracy**	79.5%<br>[73.9 ; 85.1]%<br>*p = 0.112*	71.5%<br>[65.2 ; 77.8]%<br>***p = 0.009***	84.0%<br>[78.9 ; 89.1]%<br>*p = 0.674*	82.0%<br>[76.7 ; 87.3]%<br>*p = 0.895*	73.5%<br>[67.4 ; 79.6]%<br>***p = 0.003***	71.0%<br>[64.7 ; 77.3]%<br>***p = 0.006***	82.5%<br>[77.2 ; 87.8]%<br>*p = 0.410*	79.0%<br>[73.4 ; 84.6]%<br>*p = 0.372*	86.0%<br>[81.2 ; 90.9]%	83.0%<br>[77.8 ; 88.2]%
**Positive predictive value (PPV)**	54.1%<br>[42.7 ; 65.4]%<br>***p = 0.005***	62.5%<br>[54.6 ; 70.4]%<br>***p < 0.001***	67.4%<br>[53.4 ; 81.4]%<br>*p = 0.110*	74.8%<br>[66.8 ; 82.7]%<br>*p = 0.096*	46.6%<br>[36.2 ; 57.0]%<br>***p < 0.001***	61.7%<br>[53.9 ; 69.5]%<br>***p < 0.001***	62.5%<br>[48.8 ; 76.2]%<br>***p = 0.044***	70.7%<br>[62.7 ; 78.8]%<br>***p = 0.022***	88.0%<br>[75.3 ; 100.0]%	85.5%<br>[78.0 ; 93.1]%
**Negative predictive value (NPV)**	94.4%<br>[90.4 ; 98.4]%<br>***p = 0.025***	94.6%<br>[88.7 ; 100.0]%<br>***p = 0.034***	88.5%<br>[83.6 ; 93.5]%<br>*p = 0.548*	91.8%<br>[85.9 ; 97.6]%<br>*p = 0.056*	94.6%<br>[90.5 ; 98.8]%<br>***p = 0.029***	98.0%<br>[94.2 ; 100.0]%<br>***p = 0.007***	88.8%<br>[83.8 ; 93.8]%<br>*p = 0.502*	92.2%<br>[86.2 ; 98.2]%<br>*p = 0.054*	85.7%<br>[80.5 , 90.9]%	81.2%<br>[74.1 ; 88.3]%
**Positives (alert rate) in clinical cohort (n,%)** – **outpatients**	1,305 (36.1%)		717 (19.8%)		1,592 (44.0%)		816 (22.5%)		385 (10.6%)	
**inpatients**	8,427 (70.8%)		6,688 (56.2%)		8,957 (75.3%)		7,037 (59.1%)		4,836 (40.6%)	
**overall cohort**	9,884 (62.6%)		7,517 (47.6%)		10,713 (67.9%)		7,975 (50.5%)		5,303 (33.6%)	

Table S1 - Estimated Metrics in Clinical Cohort with Optimized / AI Developer's Threshold in Pathology Pleural Effusion. Given metrics are derived from expected prevalence according to results of a random sample reading of 200 cases respectively from the inpatient and outpatient group (see Table 1). 95% confidence intervals are shown in square brackets []. The reported p-values refer to comparisons of differences with the AI Developer Thresholds. The thresholds used and statistically significant p-values are highlighted in bold.

**Reference standard / algorithm versions**	**RFS III**				**RFS IV**				**algorithm version 10**	
**Threshold optimized for**	**Outpatient group (OPOT)**		**Inpatient group (IPOT)**		**Outpatient group (OPOT)**		**Inpatient group (IPOT)**		**AI developer's threshold (AIDT)**	
**Metrics in group**	**outpatients**	**inpatients**	**outpatients**	**inpatients**	**outpatients**	**inpatients**	**outpatients**	**inpatients**	**outpatients**	**inpatients**
**Threshold**	**0.051**		**0.104**		**0.044**		**0.090**		**0.185**	
**Sensitivity**	91.5%<br>[83.5 ; 99.5]%<br>***p < 0.001***	94.8%<br>[89.1 ; 100.0]%<br>***p < 0.001***	76.6%<br>[64.5 ; 88.7]%<br>***p = 0.031***	81.0%<br>[70.9 ; 91.1]%<br>***p = 0.015***	97.9%<br>[93.7 ; 100.0]%<br>***p < 0.001***	96.5%<br>[91.9 ; 100.0]%<br>***p < 0.001***	80.9%<br>[69.6 ; 92.1]%<br>***p = 0.008***	82.8%<br>[73.0 ; 92.5]%<br>***p = 0.008***	53.2%<br>[38.9 ; 67.5]%	58.6%<br>[45.9 ; 71.3]%
**Specificity**	33.3%<br>[25.9 ; 40.8]%<br>***p < 0.001***	37.3%<br>[29.4 ; 45.3]%<br>***p < 0.001***	73.2%<br>[66.2 ; 80.2]%<br>***p = 0.004***	64.1%<br>[56.2 ; 72.0]%<br>***p < 0.001***	29.4%<br>[22.2 ; 36.6]%<br>***p < 0.001***	33.8%<br>[26.0 ; 41.6]%<br>***p < 0.001***	68.0%<br>[60.6 ; 75.4]%<br>***p < 0.001***	58.5%<br>[50.3 ; 66.6]%<br>***p < 0.001***	86.9%<br>[81.6 ; 92.3]%	85.2%<br>[79.4 ; 91.0]%
**Accuracy**	47.0%<br>[40.1 ; 53.9]%<br>***p < 0.001***	54.0%<br>[47.1 ; 60.9]%<br>***p < 0.001***	74.0%<br>[67.9 ; 80.1]%<br>*p = 0.289*	69.0%<br>[62.6 ; 75.4]%<br>*p = 0.071*	45.5%<br>[38.6 ; 52.4]%<br>***p < 0.001***	52.0%<br>[45.1 ; 58.9]%<br>***p < 0.001***	71.0%<br>[64.7 ; 77.3]%<br>*p = 0.083*	65.5%<br>[58.9 ; 72.1]%<br>***p = 0.011***	79.0%<br>[73.4 ; 84.6]%	77.5%<br>[71.7 ; 83.3]%
**Positive predictive value (PPV)**	29.7%<br>[22.2 ; 37.1]%<br>***p = 0.003***	38.2%<br>[30.3 ; 46.1]%<br>***p = 0.005***	46.8%<br>[35.6 ; 57.9]%<br>*p = 0.453*	48.0%<br>[38.1 ; 57.9]%<br>*p = 0.139*	29.9%<br>[22.6 ; 37.1]%<br>***p = 0.003***	37.3%<br>[29.6 ; 45.1]%<br>***p = 0.003***	43.7%<br>[33.3 ; 54.1]%<br>*p = 0.266*	44.9%<br>[35.4 ; 54.3]%<br>*p = 0.060*	55.6%<br>[41.0 ; 70.1]%	61.8%<br>[49.0 ; 74.7]%
**Negative predictive value (NPV)**	92.7%<br>[85.9 ; 99.6]%<br>*p = 0.271*	94.6%<br>[88.7 ; 100.0]%<br>*p = 0.063*	91.1%<br>[86.0 ; 96.1]%<br>*p = 0.247*	89.2%<br>[83.2 ; 95.2]%<br>*p = 0.274*	97.8%<br>[93.6 ; 100.0]%<br>***p = 0.047***	96.0%<br>[90.6 ; 100.0]%<br>***p = 0.044***	92.0%<br>[87.0 ; 97.0]%<br>*p = 0.167*	89.2%<br>[83.0 ; 95.5]%<br>*p = 0.290*	85.8%<br>[80.3 ; 91.3]%	83.4%<br>[77.4 , 89.5]%
**Positives (alert rate) in clinical cohort (n,%)** – **outpatients**	2,317 (64.0%)		1,268 (35.0%)		2,530 (69.9%)		1,458 (40.3%)		657 (18.2%)	
**inpatients**	8,898 (74.8%)		6,170 (51.8%)		9,365 (78.7%)		6,759 (56.8%)		4,018 (33.8%)	
**overall cohort**	11,376 (72.1%)		7,535 (47.7%)		12,073 (76.5%)		8,325 (52.7%)		4,731 (30.0%)	

Table S2 - Estimated Metrics in Clinical Cohort with Optimized / AI Developer's Threshold in Pathology Consolidation Suspicious for Pneumonia. Given metrics are derived from expected prevalence according to results of a random sample reading of 200 cases respectively from the inpatient and outpatient group (see Table 1). 95% confidence intervals are shown in square brackets []. The reported p-values refer to comparisons of differences with the AI Developer Thresholds. The thresholds used and statistically significant p-values are highlighted in bold.

Reference standard / algorithm versions		RFS III				RFS IV				algorithm version 10	
Threshold optimized for		Outpatient group (OPOT)		Inpatient group (IPOT)		Outpatient group (OPOT)		Inpatient group (IPOT)		AI developer's threshold (AIDT)	
Metrics in group		outpatients	inpatients	outpatients	inpatients	outpatients	inpatients	outpatients	inpatients	outpatients	inpatients
Threshold		**0.083**		**0.222**		**0.079**		**0.191**		**0.208**	
Sensitivity		80.0% [44.9 ; 100.0]% *p = 1*	92.0% [81.4 ; 100.0]% *p = 0.247*	80.0% [44.9 ; 100.0]% *p = 1*	76.0% [59.3 ; 92.7]% *p = 1*	80.0% [44.9 ; 100.0]% *p = 1*	96.0% [88.3 ; 100.0]% *p = 0.103*	80.0% [44.9 ; 100.0% *p = 1*	76.0% [59.3 ; 92.7]% *p = 1*	80.0% [44.9 ; 100.0]%	76.0% [59.3 ; 92.7]%
Specificity		76.4% [70.5 ; 82.4]% ***p < 0.001***	72.0% [65.3 ; 78.7]% ***p < 0.001***	96.9% [94.5 ; 99.3]% *p = 1*	90.9% [86.6 ; 95.1]% *p = 1*	74.4% [68.2 ; 80.5]% ***p < 0.001***	71.4% [64.7 ; 78.1]% ***p < 0.001***	95.4% [92.4 ; 98.3]% *p = 0.798*	89.7% [85.2 ; 94.2]% *p = 1*	96.4% [93.8 ; 99.0]%	90.3% [85.9 ; 94.7]%
Accuracy		76.5% [70.6 ; 82.4]% ***p < 0.001***	74.5% [68.5 ; 80.5]% ***p < 0.001***	96.5% [94.0 ; 99.0]% *p = 1*	89.0% [84.7 ; 93.3]% *p =1*	74.5% [68.5 ; 80.5]% ***p < 0.001***	74.5% [68.7 ; 78.1]% ***p < 0.001***	95.0% [92.0 ; 98.0]% *p = 0.809*	88.0% [83.5 ; 92.5]% *p = 1*	96.0% [93.3 ; 98.7]%	88.5% [84.1 ; 92.9]%
Positive predictive value (PPV)		8.0% [0.5 ; 15.5]% ***p = 0.042***	31.9% [21.2 ; 42.7]% *p = 0.060*	40.0% [9.6 ; 70.4]% *p = 1*	54.3% [37.8 ; 70.8]% *p = 1*	7.4% [0.4 ; 14.4]% ***p = 0.031***	32.4% [21.8 ; 43.1]% *p = 0.065*	30.8% [5.7 ; 55.9]% *p = 1*	51.4% [35.2 ; 67.5]% *p = 1*	36.4% [7.9 ; 64.8]%	52.8% [36.5 ; 69.1]%
Negative predictive value (NPV)		99.3% [98.0 ; 100.0]% *p =1*	98.4% [96.3 ; 100.0]% *p = 0.467*	99.5% [98.4 ; 100.0]% *p =1*	96.4% [93.5 ; 99.2]% *p = 1*	99.3% [98.0 ; 100.0]% *p = 1*	99.2% [97.7 ; 100.0]% *p = 0.234*	99.5% [98.4 ; 100.0]% *p = 1*	96.3% [93.4 ; 99.2]% *p = 1*	99.5% [98.4 ; 100.0]%	96.3% [93.5 ; 99.2]%
Positives (alert rate) in clinical cohort (n,%)	outpatients	700 (19.3%)		144 (4.0%)		740 (20.4%)		191 (5.3%)		167 (4.6%)	
	inpatients	3,851 (32.4%)		1,353 (11.4%)		3,995 (33.5%)		1,653 (13.9%)		1,479 (12.4%)	
	overall cohort	4,624 (29.3%)		1.522 (9.6%)		4,813 (30.5%)		1,875 (11.9%)		1,673 (10.6%)	

Table S3 - Estimated Metrics in Clinical Cohort with Optimized / AI Developer's Threshold in Pathology Pneumothorax. Given metrics are derived from expected prevalence according to results of a random sample reading of 200 cases respectively from the inpatient and outpatient group (see Table 1). 95% confidence intervals are shown in square brackets []. The reported p-values refer to comparisons of differences with the AI Developer Thresholds. The thresholds used and statistically significant p-values are highlighted in bold. Values highlighted with a gray background are subject to low statistical power due to low prevalence in the outpatient sample reading.

Reference standard / algorithm versions		RFS III				RFS IV				algorithm version 10	
Threshold optimized for		Outpatient group<br>(OPOT)		Inpatient group<br>(IPOT)		Outpatient group<br>(OPOT)		Inpatient group<br>(IPOT)		AI developer's<br>threshold (AIDT)	
Metrics in group		outpatients	inpatients	outpatients	inpatients	outpatients	inpatients	outpatients	inpatients	outpatients	inpatients
Threshold		0.030		0.251		0.022		0.186		0.089	
Sensitivity		93.8%<br>[81.9 ; 100.0]%<br><br>*p = 0.593*	100.0%<br>[100.0 ; 100.0]%<br><br>*p = 1*	68.8%<br>[46.0 ; 91.5]%<br><br>*p = 0.683*	90.9%<br>[73.9 ; 100.0]%<br><br>*p = 1*	93.8%<br>[81.9 ; 100.0]%<br><br>*p = 0.593*	100.0%<br>[100.0 ; 100.0]%<br><br>*p = 1*	68.8%<br>[46.0 ; 91.5]%<br><br>*p = 0.683*	90.9%<br>[73.9 ; 100.0]%<br><br>*p = 1*	81.3%<br>[62.1 ; 100.0]%	90.9%<br>[73.9 ; 100.0]%
Specificity		40.2%<br>[33.1 ; 47.3]%<br><br>***p < 0.001***	38.1%<br>[31.2 ; 45.0]%<br><br>***p < 0.001***	92.4%<br>[88.6 ; 96.2]%<br><br>***p < 0.001***	87.8%<br>[83.2 ; 92.5]%<br><br>***p < 0.001***	32.6%<br>[25.8 ; 39.4]%<br><br>***p < 0.001***	29.6%<br>[23.1 ; 36.1]%<br><br>***p < 0.001***	87.5%<br>[82.7 ; 92.3]%<br><br>***p = 0.019***	82.0%<br>[76.5 ; 87.5]%<br><br>***p = 0.003***	77.7%<br>[71.7 ; 83.7]%	68.3%<br>[61.6 ; 74.9]%
Accuracy		44.5%<br>[37.6 ; 51.4]%<br><br>***p < 0.001***	41.5%<br>[34.7 ; 48.3]%<br><br>***p < 0.001***	90.5%<br>[86.4 ; 94.6]%<br><br>***p < 0.001***	88.0%<br>[83.5 ; 92.5]%<br><br>***p < 0.001***	37.5%<br>[30.8 ; 44.2]%<br><br>***p < 0.001***	33.5%<br>[27.0 ; 40.0]%<br><br>***p < 0.001***	86.0%<br>[81.2 ; 90.8]%<br><br>*p = 0.051*	82.5%<br>[77.2 ; 87.8]%<br><br>***p = 0.003***	78.0%<br>[72.3 ; 83.7]%	69.5%<br>[63.1 ; 75.9]%
Positive predictive value (PPV)		12.0%<br>[6.3 ; 17.7]%<br><br>*p = 0.069*	8.6%<br>[3.7 ; 13.4]%<br><br>*p = 0.316*	44.0%<br>[24.5 ; 63.5]%<br><br>*p = 0.126*	30.3%<br>[14.6 ; 46.0]%<br><br>*p = 0.099*	10.8%<br>[5.6 ; 15.9]%<br><br>***p = 0.034***	7.6%<br>[3.3 ; 12.0]%<br><br>*p = 0.198*	32.4%<br>[16.6 ; 48.1]%<br><br>*p = 0.546*	22.7%<br>[10.3 ; 35.1]%<br><br>*p = 0.368*	24.1%<br>[12.7 ; 35.5]%	14.3%<br>[6.1 ; 22.5]%
Negative predictive value (NPV)		98.7%<br>[96.1 ; 100.0]%<br><br>*p = 1*	100.0%<br>[100.0 ; 100.0]%<br><br>*p = 1*	97.1%<br>[94.7 ; 99.6]%<br><br>*p = 0.921*	99.4%<br>[98.2 ; 100.0]%<br><br>*p = 1*	98.4%<br>[95.2 ; 100.0]%<br><br>*p = 1*	100.0%<br>[100.0 ; 100.0]%<br><br>*p = 1*	97.0%<br>[94.4 ; 99.6]%<br><br>*p = 0.861*	99.4%<br>[98.1 ; 100.0]%<br><br>*p = 1*	97.9%<br>[95.6 ; 100.0]%	99.2%<br>[97.7 ; 100.0]%
Positives (alert rate) in clinical cohort (n,%)	outpatients	2,022 (55.9%)		417 (11.5%)		2,366 (65.4%)		545 (15.1%)		973 (26.9%)	
	inpatients	8,468 (71.2%)		1,964 (16.5%)		9,477 (79.6%)		2,613 (22.0%)		4,607 (38.7%)	
	overall cohort	10,652 (67.5%)		2,417 (15.3%)		12,033 (76.2%)		3,203 (20.3%)		5,666 (35.9%)	

Table S4 - Estimated Metrics in Clinical Cohort with Optimized / AI Developer's Threshold in Pathology Nodules. Given metrics are derived from expected prevalence according to results of a random sample reading of 200 cases respectively from the inpatient and outpatient group (see Table 1). 95% confidence intervals are shown in square brackets []. The reported p-values refer to comparisons of differences with the AI Developer Thresholds. The thresholds used and statistically significant p-values are highlighted in bold.
